# Interpreting statistical evidence with S values: an illustration with trials of goal‐directed haemodynamic therapy

**DOI:** 10.1111/anae.16701

**Published:** 2025-07-23

**Authors:** Markus Huber

**Affiliations:** ^1^ Bern University Hospital, University of Bern Bern Switzerland

Asking clinical researchers regarding their relationship with p values, the answer would likely be “it's difficult”. Despite known challenges with p values with respect to clinical utility and misinterpretations, they remain ubiquitous in the medical literature [1, 2]. In search of new ways to interpret the evidence resulting from data collection, a new statistical quantity, the S value, was proposed recently [3, 4].

The S value (also termed Shannon‐information or surprisal) is a statistic quantifying the amount of evidence against a test hypothesis, for example the null hypothesis underlying a randomised trial. It is defined as the negative logarithm of an observed p value p: s = −log_2_(p). The unit of S values are called bits when using the binary logarithm. In contrast to p values, S values have a simple and intuitive interpretation related to physical experimentation, akin to tossing a coin [3, 4, 5]. Imagine tossing a fair coin four times and getting four heads. Assuming an equal chance of heads and tails, the associated p value is $\frac{1}{2}\times \frac{1}{2}\times \frac{1}{2}\times \frac{1}{2}={\left(\frac{1}{2}\right)}^{4}$. The corresponding S value is 4 bits and represents the evidence against the test hypothesis of a fair coin. Note that the conventional p value of 0.05 corresponds to an S value of 4.3 bits. To aid interpretation of S values with physical experimentation, the S values are rounded to the nearest integer. Thus, the evidence against a test hypothesis when getting a p value of 0.05 can be compared with the situation of getting four heads (or tails) when tossing an unbiased coin four times. Here, we illustrate S values with trials investigating the possible benefits of goal‐directed haemodynamic treatment and discuss their clinical utility in interpreting conventional p values.

We used published data from a previous meta‐analysis comparing goal‐directed haemodynamic treatment with control treatment [6]. Outcome data for: mortality; sepsis; surgical site infection; pneumonia; urinary tract infection (UTI); acute kidney injury (AKI); paralytic ileus; the need for re‐operation; and the incidence of ≥ 1 complication were available. Using published outcome data, we recalculated p values using a random‐effects model to pool the individual studies. Associated S values are computed with these recalculated p values for each outcome. All analyses were performed with R (R Foundation for Statistical Computing, Vienna, Austria).

Figure 1 displays the p and S values for the nine outcomes. The black dotted line displays the relationship from the S value's definition: s = −log_2_(p). The S values for the re‐operation, sepsis, mortality and paralytic ileus outcomes are small with zero to two bits. The evidence against the null hypothesis of no treatment benefit for the outcomes AKI and UTI are 4 bits and 5 bits, respectively. In contrast, the S values for pneumonia, ≥ 1 complication and surgical site infection are 10 bits, 19 bits and 25 bits, respectively. Table 1 provides an overview of the numeric values.

**Figure 1:**
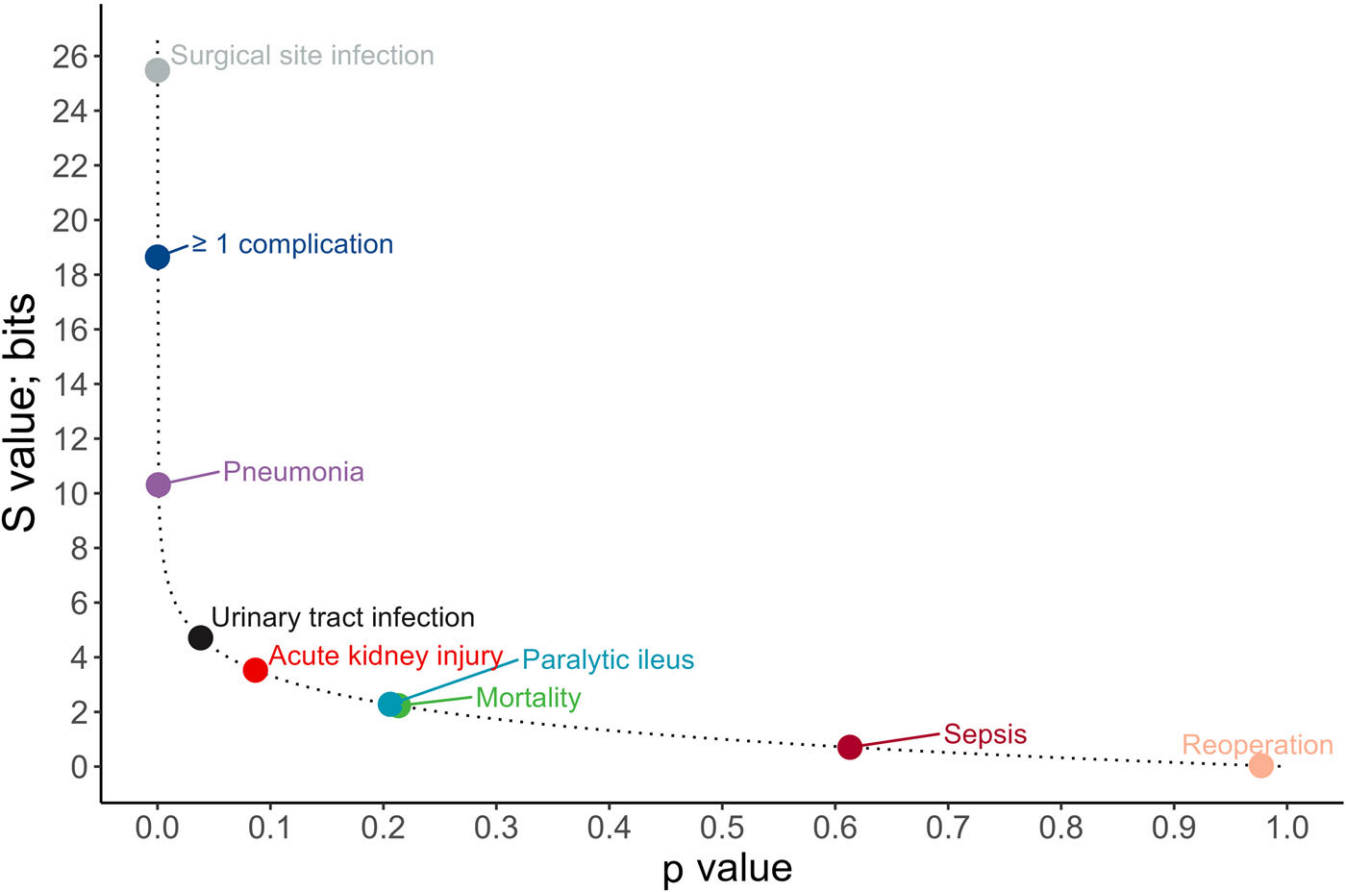
Illustration of p values and corresponding S values for a number of outcomes recalculated from a published meta‐analysis comparing goal directed haemodynamic therapy with control treatment [6]. The black dotted line represents the definition of the S values (s = −log_2_(p)).

**Table 1:** The p values and S values derived from published outcome data of a meta‐analysis comparing the treatment effects of goal‐directed haemodynamic treatment vs. control treatment [6]. The S values are computed by the negative logarithm (base 2) of the p values (s = −log_2_(p)) and provide a measure of the evidence against a test hypothesis (e.g. a null hypothesis regarding the relative efficacy of two alternative treatments in a randomised controlled trial). To aid interpretation of the S values with physical experimentation (with the repeated tossing of a fair coin) the S values are rounded to the nearest integer. For example, the S value of 10.3 can be likened to the situation when tossing a fair coin 10 times resulting in either 10 heads or 10 tails.

Outcome	p value	S value (bits)
Surgical site infection	< 0.0001	25.5
≥ 1 complication	< 0.0001	18.6
Pneumonia	0.0008	10.3
Urinary tract infection	0.038	4.7
Acute kidney injury	0.087	3.5
Paralytic ileus	0.206	2.3
Mortality	0.213	2.2
Sepsis	0.613	0.7
Re‐operation	0.977	0.03

The non‐linear relationship between S and p values has important implications with respect to interpretation when comparing two p values. For example, the p values for sepsis (p = 0.61) and mortality (p = 0.21) differ by 0.4 but differ in their amount of evidence against the null hypothesis only by 1 bit. In contrast, the p values for UTI (p = 0.038) and pneumonia (p = 0.0008) differ by only 0.037, which is a magnitude smaller. However, these two outcomes differ in their S values by 5 bits – a rather large difference. This difference corresponds to the probability of getting five heads when tossing a fair coin five times vs. the scenario of getting 10 heads when tossing an unbiased coin 10 times.

Overall, there are an abundant number of cases in the medical literature where treatment benefits are evaluated only with respect to the conventional significance level of 0.05. The proposed S value does not aim to replace the traditional p value. However, its aim is to provide a more intuitive interpretation of the collected evidence against a test hypothesis. Additionally, S values help interpreting different p values. Imagine two outcomes with corresponding p values of p = 0.049 and p = 0.051, respectively. The evidence against the test hypothesis is 4.4 bits and 4.3 bits, respectively. In the context of the coin‐tossing experiment, the evidence against the test hypothesis would be the same in these cases, which questions the practice of dichotomising the evidence into being either significant or non‐significant.

I have shown that S values provide an intuitive interpretation of the pooled evidence from multiple randomised controlled trials with respect to a treatment effect of goal‐directed haemodynamic treatment and can be compared with the situation of getting all heads (or tails) when tossing a fair coin four times (AKI); five times (UTI); 10 times (pneumonia); 19 times (≥ 1 complication); and 25 times (surgical site infection). Although some studies have been analysed by means of S values [5], a broad assessment of their clinical utility and their adoption in clinical research is still missing. Yet in terms of the potential of S values to change the interpretation of evidence against a test hypothesis, Kenneth J Rothman put it succinctly: “*If the S value moves us away from focusing on significance testing, it is worth a try*” [7].

## References

[1] Greenland S , Senn SJ , Rothman KJ , Carlin JB , Poole C , Goodman SN , Altman DG . Statistical tests, p values, confidence intervals, and power: a guide to misinterpretations. Eur J Epidemiol 2016; 31: 337–350. 10.1007/s10654-016-0149-3. 27209009 PMC4877414

[2] Matthews R . The p‐value statement, five years on. Significance 2021; 18: 16–19. 10.1111/1740-9713.01505.33821160

[3] Greenland S . Valid p‐values behave exactly as they should: some misleading criticisms of p‐values and their resolution with S‐values. Am Stat 2019; 73(Supp 1): 106–114. 10.1080/00031305.2018.1529625.

[4] Rafi Z , Greenland S . Semantic and cognitive tools to aid statistical science: replace confidence and significance by compatibility and surprise. BMC Med Res Methodol 2020; 20: 244. 10.1186/s12874-020-01105-9. 32998683 PMC7528258

[5] Mansournia MA , Nazemipour M , Etminan M . *P*‐value, compatibility, and *S*‐value. Glob Epidemiol 2022; 4: 100085. 10.1016/j.gloepi.2022.100085. 37637018 PMC10446114

[6] Jalalzadeh H , Hulskes RH , Weenink RP , et al. Systematic review and meta‐analysis of goal‐directed haemodynamic therapy algorithms during surgery for the prevention of surgical site infection. eClin Med 2024; 78: 102944. 10.1016/j.eclinm.2024.102944. PMC11647171 39687427

[7] Rothman KJ . Rothman responds to “surprise!”. Am J Epidemiol 2021; 190: 194–195. 10.1093/aje/kwaa137. 33524113

